# Subgroup Splits in Diverse Work Teams: Subgroup Perceptions but Not Demographic Faultlines Affect Team Identification and Emotional Exhaustion

**DOI:** 10.3389/fpsyg.2021.595720

**Published:** 2021-02-12

**Authors:** Kevin E. Tiede, Stefanie K. Schultheis, Bertolt Meyer

**Affiliations:** ^1^Graduate School of Decision Sciences and Department of Psychology, University of Konstanz, Konstanz, Germany; ^2^Department of Psychology, Ruhr University Bochum, Bochum, Germany; ^3^Department of Psychology, Chemnitz University of Technology, Chemnitz, Germany

**Keywords:** faultlines, subgroup perception, team identification, subgroup identification, emotional exhaustion

## Abstract

We investigate the relationship between (hypothetical) subgroup splits (i.e., faultlines), subjectively perceived subgroups, and team identification and emotional exhaustion. Based on the job demands-resources model and on self-categorization theory, we propose that faultline strength and perceived subgroups negatively affect emotional exhaustion, mediated by team identification. We further propose that subgroup identification moderates the mediation such that subgroup identification compensates low levels of team identification. We tested our hypotheses with a two-wave questionnaire study in a sample of 105 participants from 48 teams from various contexts. We found an effect of perceived subgroups on emotional exhaustion mediated by team identification, but no direct or indirect effect of faultline strength on emotional exhaustion. We also could not find that subgroup identification moderates the effect of team identification on emotional exhaustion. We discuss the need for further research on the link of subgroup splits in work teams and the rise of psychological health issues and derive that measures to prevent burnout should primarily focus on avoiding or reducing subgroup perception whereas affecting the actual demographic composition of work team should be of lower priority.

## Introduction

The number of days of absence due to psychological health issues in Germany has more than tripled between 1997 and 2018 (DAK-Gesundheit, 2019). In 2015, psychological health issues led to an estimated loss of about 44.4 billion Euro for the German economy (Bundesanstalt für Arbeitsschutz und Arbeitsmedizin, 2018). Therefore, psychological health issues at the workplace have become a major concern for employees and employers.

Even though burnout, conceptualized as “a prolonged response to chronic emotional and interpersonal stressors on the job, […] comprised by the three dimensions of exhaustion, cynicism, and inefficacy” (Maslach et al., 2001, p. 397), is not yet recognized as a mental illness by the two major classification systems ICD-10 and DSM-V, research on the causes of burnout is clearly important because burnout is evidently related to depression (Ahola et al., 2005). Depression, in turn, is responsible for the majority of days absent due to psychological health issues (Knieps and Pfaff, 2016; DAK-Gesundheit, 2019).

As the roots of burnout can, by definition, be found in the workplace, its prevention must also be tied to the workplace. Attempts to identify antecedents of burnout often use the job demands-resources model of burnout (Demerouti et al., 2001) and focus on job demands such as time pressure, physical, emotional, or cognitive demands (see Bakker and Demerouti, 2007, for a review). However, we propose that subgroup structures in work teams may also play a crucial role in explaining the rise in burnout. Subgroups are “subsets of team members that are each characterized by a unique form or degree of interdependence” (Carton and Cummings, 2012, p. 441). More precisely, we propose that strong faultlines and the perception of subgroups are additional job demands emerging from (demographic) changes in the workforce. Faultlines are hypothetical dividing lines that split a group into subgroups based on one or more individual attributes (Lau and Murnighan, 1998). While faultlines are based on objective attributes, it is also important to understand the role of subjective perceptions of subgroup structure. By investigating the link between faultlines/perceived subgroups and burnout, we aim to capture the effects of multiple major trends that contribute to the diversification of the workforce. For example, the age distribution of the workforce and other corresponding attributes, like values and expectations, shifted dramatically (Burke and Ng, 2006). Apart from that, globalization has led to a global workforce (Burke and Ng, 2006), bringing along further diversity. Moreover, in the long run, the recent flow of refugees in many parts of the world will further intensify the current level of diversification of ethnicity, religion, and the nature of prior work experience and education. In sum, those trends lead toward greater levels of diversity in work teams.

Previous studies on the relationship between team diversity and psychological well-being yielded mixed results: In a sample of predominantly female federal tax officer teams, team age diversity was positively correlated with health issues, but team gender diversity was negatively correlated with health issues (Wegge et al., 2008). In another study with warehouse workers, moderate levels of racial/ethnic similarity were associated with less lumbar back health problems (Hoppe et al., 2014). However, this study revealed opposing effects for different ethnic groups: Latino and African-American employees working in groups with high racial/ethnic similarity, reported less health problems than groups with low racial/ethnic similarity, but among white employees, high racial/ethnic similarity was associated with more health problems. Overall, no clear pattern can be found in the results obtained up to now.

Consequently, further research needs to clarify, if and under which circumstances diversity and group composition affect well-being. Therefore, the aim of our study is to extend the current research on diversity as a job demand that may cause health problems and, by doing so, to overcome some of its shortcomings. Specifically, we investigate whether stronger faultlines and stronger perceptions of subgroups are related to higher levels of burnout, instead of looking at different forms of diversity separately.

As opposed to diversity which is known to yield mixed results, strong faultlines are more consistently associated with negative outcomes (Thatcher and Patel, 2012; Meyer, 2017). Hence, we assume that faultlines will also lead to more consistent results in the domain of well-being. In contrast to diversity measures, faultlines consider the distribution of multiple attributes simultaneously. This approach is more promising because it allows to capture the multiple ways of increasing diversity we outlined above all in one. It enables us to account for the joint effects that occur when a team faces diversity in more than one attribute (e.g., age and educational background). Previous research in other contexts has successfully demonstrated that faultlines explain more variance than single-attribute heterogeneity indexes (Lau and Murnighan, 2005).

Based on faultline theory’s theoretical underpinnings, self-categorization theory (Turner et al., 1987) and social identity theory (Tajfel and Turner, 1986), we argue that team identification is a mediator in the positive association of faultlines and burnout: In-group and out-group perception and the resulting intergroup bias (Tajfel and Turner, 1986) are detrimental to team identification, which, in turn, is beneficial for well-being (Onyett et al., 1997; Haslam et al., 2005, 2009; Cicero et al., 2007; Jimmieson et al., 2010).

We further propose that splits into subgroups are especially detrimental for employee psychological health if employees subjectively perceive them. While demographic faultlines are situated on a hypothetical level and are based on objective attributes, the perception of subgroups constitutes their subjective counterpart. Considering subjective perceptions, too, is useful because objective and subjective measures yielded different results in some studies on diversity (e.g., Hentschel et al., 2013) and faultlines (Jehn and Bezrukova, 2010; Thatcher and Patel, 2012). In this way, our study can also help to clarify, if the actual group composition or rather the (sub)group perception, or both, influence well-being in terms of burnout.

Because team members may not only identify with the whole team but also with their subgroup, furthermore, we consider subgroup identification as a moderator of the relationship between team identification and burnout. Assuming that team identification mitigates burnout (Onyett et al., 1997; Haslam et al., 2009), we argue that identification with a part of the team can be beneficial, too.

In sum, our study makes several contributions to research on psychological health issues and diversity research: First of all, this study examines faultlines as precedent of emotional exhaustion. So far, research on the effect of faultlines on psychological health is scarce. By including team identification as a mediator in our model, we propose a mechanism for the effect of faultlines on emotional exhaustion. Second, we investigate objective faultlines and subjectively perceived subgroups as well as their unique contribution to the proposed effect. This enables us to identify whether both, objective and subjective variables, are important and if so, to compare their impact on psychological health. Third, this study investigates the attenuating effect of subgroup identification on the consequences of lacking team identification. Not only does this study look at a moderator of this effect, but it also conceptualizes team identification not as the opposite of subgroup identification but two independent constructs.

## Theoretical Background

Burnout consists of the three facets emotional exhaustion, cynicism, and inefficacy (Maslach et al., 2001)^1^. Out of the three dimensions, emotional exhaustion is the central aspect of burnout (Maslach et al., 2001). It involves feelings of being emotionally overcharged and “depleted of one’s emotional and physical resources” (Maslach and Leiter, 2008, p. 498; Maslach and Jackson, 1981). Due to its well-researched underpinnings, we decided to focus on emotional exhaustion exclusively. In addition, emotional exhaustion is the focus of many studies on psychological health in the work-related context (Maslach and Leiter, 2008).

As mentioned above, the job demands-resources model of burnout is a useful framework for analyzing the causes of burnout. It classifies characteristics of work into demands and resources. Demands foster burnout while resources may help to prevent the development of burnout (Demerouti et al., 2001). Although physical, psychological, social, and organizational factors can all reflect job demands, previous research on the job demands-resources model has mainly focused on demands directly tied to the worker’s role and tasks such as role ambiguity, role conflict, workload, and time pressure (for meta-analyses, see Crawford et al., 2010; Alarcon, 2011). Although social factors have been studied as resources (Nahrgang et al., 2011), to the best of our knowledge there has been no study which investigated the structure of the work team as either demand or resource. In our study, we assume that increasing diversity in work teams can be challenging for their members, as it may facilitate splits into subgroups. More precisely, we interpret faultline strength and the perception of subgroups as job demands. Furthermore, we want to meet the recent calls for using multilevel approaches to overcome the focus on either individual-level or team-level demands only (Bakker and Demerouti, 2017). Thus, our study contributes to the knowledge of the job demands-resources model by examining group structure as a job demand simultaneously on the individual level (i.e., perceived subgroups) and the team level (i.e., faultlines).

### Faultlines

The faultline model posits that various diversity attributes can be aligned so that relatively homogeneous subgroups emerge which are delineated by hypothetical dividing lines, the so-called faultlines (Lau and Murnighan, 1998; Thatcher and Patel, 2012). Thus, the faultline model considers the effect of multiple attributes and their interrelationships on potential subgroup formation at the same time (Lau and Murnighan, 1998). As multiple forms of diversity occur simultaneously in work teams, the faultline model captures the reality of work teams more closely than the investigation of single diversity attributes: For example, imagine a team of four female American lawyers who all studied in Harvard. Two of them are 30, while the other two are aged 58 and 61. In this group, age clearly separates the group into two subgroups. Imagine a second team of four lawyers: One is male, Chinese, 30, and studied at Yale; one is female, American, 30, and studied at Harvard; one is female, Chinese, 58, and studied at Princeton; one is female, American, 61, and studied at Yale. Although both teams have the same level of age diversity, age diversity only is a strongly separating factor in the first team, but not in the second team.

Strong faultlines result from high correlations among attributes that reflect the alignment of multiple differences. This alignment makes actual splits into subgroups more likely; or in other words: The higher the correlation of attributes, the more homogenous subgroups emerge, making the faultline stronger (Lau and Murnighan, 1998).

Most faultline computations capture the meta-contrast ratio, which reflects the ratio of average inter-category differences to average intra-category differences (Turner, 1985). Faultlines are a hypothetical construct that does not necessarily align with real-world splits into subgroups. However, faultline theory builds on self-categorization theory and social identity theory which explain why differences between team members (or other groups) are likely to result in perceived subgroup splits.

Self-categorization theory postulates that individuals seek maximum self-esteem through comparing themselves with others, a process called social categorization (Turner et al., 1987). It explains how people see themselves and others as parts of groups depending on social categories, which in turn can lead to a perception of an in-group and an out-group (Tajfel and Turner, 1986). After having categorized themselves using salient social attributes, individuals are prone to regard others who they perceive to be the most similar to themselves as part of their in-group. According to social identity theory, so-called intergroup bias leads to a less favorable evaluation of someone perceived as an out-group member compared to in-group members (Tajfel and Turner, 1986). Unfavorable evaluations of some team members may lead to a number of negative outcomes, such as increased conflict, less cohesion, less trust, and less knowledge exchange (van Knippenberg and Schippers, 2007). In line with these findings, overall, faultlines research revealed primarily negative consequences of strong faultlines on team processes and team-level outcomes (for a review, see Thatcher and Patel, 2012). Therefore, we expect faultlines to be detrimental for well-being as well.

All of the known negative outcomes of faultlines may potentially act as mediators in the relationship between faultline strength and emotional exhaustion, with the mediator being an immediate result of faultlines that manifests in higher emotional exhaustion over time. Despite it being less established in previous studies, we propose team identification is a crucial mediator in this relationship. Team identification is the emotional significance team members attach to their membership in a certain team (van der Vegt and Bunderson, 2005). Two studies did not find any significant correlation between faultlines and team identification when originally examining team identification as a moderator (Bezrukova et al., 2009; Jehn and Bezrukova, 2010). However, these null-effects were based on zero-order correlations without any other predictors in a model. In contrast to this finding, self-categorization theory and social identity theory support the idea that strong faultines decrease team identification: a sense of social belonging as captured in team identification is a core element of these theories. Moreover, as the motive behind categorization is maximizing self-esteem (Turner et al., 1987), and as intergroup bias occurs (Tajfel and Turner, 1986), the existence of strong faultlines, or a high likelihood of splits into in-groups and out-groups, should increase the importance of in-group membership and decrease the emotional significance attached to team membership on the whole-team level. Therefore, this study also aims to provide further empirical evidence on the effect of faultlines on team identification.

Beyond that, we chose to investigate team identification as a mediator, because of the existing evidence for a link between team identification and burnout (i.e., emotional exhaustion). Team identification has positive effects on well-being in general as well as curative effects on burnout in particular (Onyett et al., 1997; Haslam et al., 2005, 2009; Cicero et al., 2007; Jimmieson et al., 2010). Another study in a longitudinal design even showed that the initial level of team identification had a curative effect on burnout in a later time point when the group was exposed to great strains (Haslam et al., 2009).

Therefore, we propose a positive association of faultline strength on burnout, mediated by team identification:

Hypothesis 1a: Faultline strength is positively associated with emotional exhaustion, mediated by team identification: The stronger the faultline, the lesser members identify with their team; the lesser team members identify with their team, the more they experience emotional exhaustion.

We note our hypothesis stands in contrast to the attenuating effect of strong faultlines on the relationship between perceived interpersonal injustice on the one hand and anxiety and depression on the other (Bezrukova et al., 2010). However, this finding only seem contradictory to our predictions at first sight. Bezrukova et al. (2010) investigated faultlines as a moderator for very specific situations of injustice and found subgroup cooperation to be the mechanism behind the beneficial effects of faultlines. We account for potential positive effects like Bezrukova et al. (2010) by incorporating subgroup identification into our model – a fact that we will turn to in detail below. In contrast to their study, we take a more general perspective in our study and investigate faultlines as a direct predictor of health without limiting its effects to certain conditions (like perceived interpersonal injustice in Bezrukova et al., 2010).

### Perceptions of Subgroups and Emotional Exhaustion

So far, our arguments focus on the role of objective faultlines in work teams. Although algorithms to measure objective, often demographic, faultlines demonstrably perform well when predicting team variables (Meyer and Glenz, 2013), these measures do not necessarily reflect the subjectively perceived subgroup structure (Homan et al., 2010; Jehn and Bezrukova, 2010; Meyer et al., 2011), i.e., the salience of social categories that faultlines are assumed to increase (Meyer et al., 2011). That is why some faultline scholars argue that for having an effect, hypothetical dividing lines (i.e., objective or dormant faultlines) must have an actual bearing on perceptions and interdependence (i.e., they must be subjectively perceived or active; Jehn and Bezrukova, 2010; Carton and Cummings, 2012). However, objective faultlines can have consequences even when they are not perceived (Chrobot-Mason et al., 2009). Accordingly, an objective faultline measure would not be able to capture the effect of subgroup structure on emotional exhaustion if the relationship hinges on the perception of subgroups rather than on potential subgroup splits as captured by objective dormant faultline measures. This issue makes it necessary to disentangle objective or dormant faultlines and perceived or active subgroups in order to investigate whether potential subgroup structures, their perception, or both affect emotional exhaustion. Therefore, we intend to shed light onto (a) the effect of perceived subgroups on emotional exhaustion via team identification and (b) the unique variances explained by objective faultlines and subjective subgroup splits.

Perceived subgroups might influence emotional exhaustion more strongly than dormant faultlines, because social categorization processes hinge on perceiving differences in the first place (Turner et al., 1987). Therefore, the perception of faultlines and subgroups should lead to the negative effects proposed by self-categorization theory. Furthermore, other theories which have been proposed to explain faultline processes such as distance theory (Brewer et al., 1993) rely on subjective perceptions as well (Meyer, 2017). Indeed, perceived faultlines are more strongly related to intersubgroup conflict than objective faultlines (Greer and Jehn, 2007). Another study found that stronger perceptions of subgroups lead to stronger coalition formation and conflict which, in turn, lead to lower levels of group outcomes and satisfaction (Jehn and Bezrukova, 2010). This study also showed that dormant objective faultlines and perceived activated faultlines need not be directly related. Another study also found no main effect of objective subgroup structure on its subjective perception but found an interaction of objective subgroup structure and diversity beliefs, with an effect of objective on subjective subgroup structure for those with low levels in diversity beliefs (Homan et al., 2010). In sum, the relation of objective faultlines and perceived subgroup structure is not fully understood yet (Homan et al., 2010; see also Meyer et al., 2011).

The inconsistent effects of dormant on subjective faultlines could be because demographic faultlines are not meaningful to team members. Social categorization emerges when the categorization is meaningful to the group members (van Knippenberg et al., 2004). Therefore, it is possible that hypothetical subgroup splits based on demographic variables such as gender, age, or tenure are not perceived as meaningful by the group members. In this case, they probably do not categorize the team members based on these attributes (van Knippenberg et al., 2004; Meyer et al., 2011). Rather, the team could be split up into subgroups based on other, more meaningful or task-relevant variables such as personality or skills. That way, finding no effect of (objective) faultlines does not necessarily mean that fautlines *per se* do not affect emotional exhaustion but rather that the objective faultline measure did not include all variables that were actually meaningful for subgroup formation.

In this study, we therefore examine an objective as well as subjective measure of subgroups to get further insights into the effect of subgroup splits on emotional exhaustion. This way, we want to gain knowledge on whether objective or subjective variables are more important by comparing the strength of both effects when controlled for the other predictor.

We propose the analog effects for perceived subgroups as for faultline strength. In line with self-categorization theory and previous research (Onyett et al., 1997; Haslam et al., 2005, 2009; Cicero et al., 2007; Jimmieson et al., 2010), we posit that perceived subgroups lead to higher emotional exhaustion via team identification. Therefore, we propose the following hypothesis:

Hypothesis 1b: Perceived subgroups are positively associated with emotional exhaustion, mediated by team identification: The stronger team members perceive subgroups, the lesser members identify with their team; the lesser team members identify with their team, the more they experience emotional exhaustion.

### Subgroup Identification as a Moderator

So far, we have proposed negative effects of faultlines: Stronger faultlines lead to higher levels of emotional exhaustion due to lower levels of team identification. Taking a less pessimistic view on faultlines, we propose that some scenarios can reverse or at least attenuate this negative effect. Identification with a group of people has a positive effect on emotional exhaustion (Haslam et al., 2005). Social identity theory (Tajfel and Turner, 1986) argues that stronger in-groups and out-groups lead to less identification with the whole team, but also emphasizes an increased identification with the subgroup. When team identification has a positive effect on well-being, we assume that identification with a particular subgroup can elicit similar positive effects. By incorporating subgroup identification as a moderator, we also account for the mentioned mediation effect of subgroup cooperation in the interactive effect of perceived interpersonal injustice and faultlines on anxiety and depression: Subgroup cooperation was the mechanism of action leading to lower levels of anxiety and depression when perceived interpersonal injustice was high and when faultlines were strong (Bezrukova et al., 2010). Cooperation in teams and team identification are closely linked (Lin et al., 2017).

While other studies (e.g., Hentschel et al., 2013) conceptualized team identification as one end of a continuum ranging from team identification to subgroup identification, we believe that high levels of subgroup identification can occur simultaneously with high levels of team identification. Research has shown that it is possible to perceive others simultaneously as in-group and as out-group members based on multiple dimensions (Hewstone et al., 2002). This so called cross-categorization (Brewer, 2000; Crisp and Hewstone, 2000) can lead to identification with a subgroup as well as, on a higher level, the whole work team. A study on cross-categorization has shown that intergroup-bias is lowest in university students of different faculties when both university and faculty membership were made salient than when only one of the two categories was made salient (Hornsey and Hogg, 2000).

According to subgroup theory (Carton and Cummings, 2012), the subgroup structure within the work team is also important for the level of team identification: Within a group, an increasing number of subgroups who share an identity makes it harder to “sense that there is a unified whole” (p. 454), whereas an increasing variation in subgroup size decreases this identity fragmentation.

Because the two types of identification do not have to, but can occur simultaneously and both types are assumed to have positive effects on psychological well-being, in this study, we test if high levels of subgroup identification can attenuate the harmful impact of low levels of team identification on emotional exhaustion. Our study therefore extends further research by conceptualizing subgroup and team identification as two negatively correlating, but separate dimensions.

Hypothesis 2: Subgroup identification moderates the effect of team identification on emotional exhaustion. The higher subgroup identification, the weaker is the relationship between team identification and emotional exhaustion.

**FIGURE 1:**
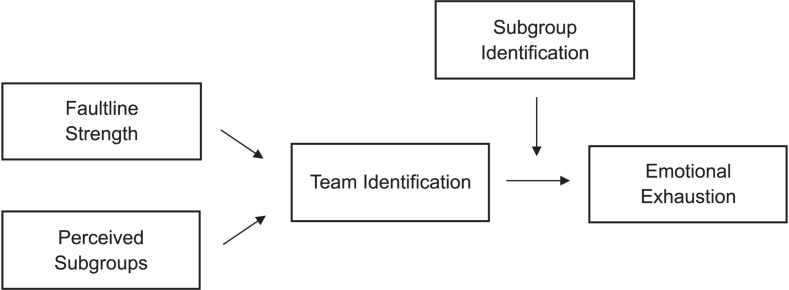
Graphical summary of our hypotheses.

Figure 1 summarizes our hypotheses. We tested them with a two-wave field study with teams from diverse backgrounds.

## Materials and Methods

### Sample and General Procedure

We gathered data at two measurement time points from members of work teams from a diverse set of backgrounds and industries to be able to draw conclusions about teams in general, going beyond a specific industry or type of team. Teams could participate when they met the criterion of two or more people who interdependently work on a task that contributes to organizational goals (Larson, 2010). In the first wave, a group of research students who participated in the design of this study for course credit distributed questionnaires in their larger personal and professional networks. At this first measurement time point, we measured faultlines and perceived subgroups as well as team and subgroup identification. At the second assessment, we asked participants to fill out a second questionnaire measuring emotional exhaustion. This design allowed us to investigate the effect of faultlines and perceived subgroups on emotional exhaustion via team identification. Data collection for this study was part of a wider research project, and some of the scales used in this study have been used in another manuscript (Schulte et al., 2020), as specified in the data transparency table in the Appendix.

In total, 536 participants from 107 teams participated in the survey in at least one wave. In the first wave, 470 participants from 106 teams returned the first questionnaire, while 178 participants from 59 teams returned the second one, with 112 individuals participating in both measurements. The average participation rate within the teams was 66.2% of all team members for the first and 56.7% for the second questionnaire. For our analysis, we included only participants who participated in both parts of the study, stated that they participated seriously and from which we had valid data for the relevant variables. Additionally, we included only groups with at least two team members. Our final data set included 105 participants from 48 teams^2^. The sample consisted of 68 women and 37 men with a mean age of 37.0 years, *SD* = 12.1. Seventy-four percent of the participants held a university degree. Participants’ reported team sizes ranged from 2 to 12 members, *M* = 5.8, *SD* = 2.7. The number of members per team who participated ranged from 1 to 7, *M* = 2.2, SD = 1.4. The teams have been working together on average for 3.2 years (SD = 3.9). The teams had diverse backgrounds: business (27.6%), public service (35.2%), police (4.8%), and volunteering (14.3%; other: 18.1%). Compared to participants who participated in the first wave only, participants who completed both waves were older, more educated, and had a slightly higher team identification. The samples did not differ in other variables (for details, see Appendix Table A2). We offered an individual team diagnosis as compensation for participation.

### Measures

#### Faultline Strength

We measured faultlines strength using the average silhouette width measure (ASW; Meyer and Glenz, 2013). ASW employs a two-stage cluster analysis approach for detecting and quantifying the subgroup structure of a group (for details and computation examples, see Meyer and Glenz, 2013): In the first step, for each team, the algorithm employs average linkage and ward clustering procedures to derive a set of possible homogeneous clusters (i.e., subgroups) of team members based on their demographic attributes that are employed for faultline detection. For all set of possible cluster solutions, the algorithm subsequently determines the degree to which each team member fits into their subgroup with the individual silhouette width (Rousseeuw, 1987). A focal individual team member’s *i* individual silhouette width *s(i)* is given as *s(i)* = *b_*i*_ – a_*i*_*/max(*a_*i*_, b_*i*_*), where *a*_*i*_ is the average distance between *i* and all other members of the same subgroup and *b*_*i*_ is the average distance between *i* and all members of the nearest different subgroup. ASW is the average value across all team members’ *s(i)* values, and the ASW faultline algorithm picks the clustering solution with the highest ASW value, which it outputs as a measure of faultline strength. Thus, the ASW value, ranging from 0 to 1, can be interpreted as denoting the extent to which the identified subgroups are homogeneous. It can also be interpreted as quantizing how much of the variability among team members is present between subgroups. For a given team, the ASW algorithm returns the corresponding value, the number of subgroups the algorithm identified for the given team, and which team member was categorized into which subgroup.

In line with previous studies (Thatcher and Patel, 2012), we computed faultlines on the basis of gender, age, and educational background. This combination served to cover attributes that are relevant for different types of faultlines. Namely, the basic demographic information on gender and age is a social category characteristic that rather creates identity-based faultlines, while educational background is an information-based and task-relevant characteristic that results in informational faultlines (Bezrukova et al., 2009). Thus, we investigated a type of faultline that is neither purely identity-based nor purely informational. Although the hypotheses we developed are more strongly related to the concepts of identity and social categorization, our rationale behind mixing social category and informational characteristics was to acknowledge that informational characteristics are likely to influence subgroup formation as well. Furthermore, the majority of faultline studies use demographic variables including the ones used in the present study (e.g., Bezrukova et al., 2010; Zanutto et al., 2011; Meyer and Glenz, 2013), with our selected variables being the three most commonly used attributes in faultline research (Thatcher and Patel, 2012).

#### Perceived Subgroups

Due to the novel investigation of perceived subgroups and, thus, a lack of a respective questionnaire, we developed a three-item scale to measure subjective perception of existing subgroups within a work team. The three items were: “Team members who are similar to each other, consort with each other more often.”, “Often, the same conversation groups form within my team.”, and “Within my team, different subgroups have emerged, whose members get along with each other well.”. The seven-point scale ranged from “strongly disagree” to “strongly agree.” The internal consistency for this measure was satisfactory (Cronbach’s α = 0.78).

#### Team Identification

We measured team identification with three items adapted from Mullin and Hogg (1998): “I identify with all members of my team.”, “In my team, I feel attached to my whole team.”, and “In my team, I feel equally strongly connected to all team members.”. The seven-point scale ranged from “strongly disagree” to “strongly agree”. Internal consistency for this measure was satisfactory (Cronbach’s α = 0.79).

#### Subgroup Identification

To measure subgroup identification, we also developed three items based on the team identification scale. The items were developed to measure identification with a particular subgroup. The items were: “In my team, I belong to an informal subgroup.”, “In my team, I feel particularly close to a certain subgroup.”, and “When I think of my team, I think of my clique rather than the whole team.”. The 7-point scale ranged from “strongly disagree” to “strongly agree.” Internal consistency for this measure was moderate (Cronbach’s α = 0.65).

#### Emotional Exhaustion

We used the emotional exhaustion subscale of the German translation of the “Maslach Burnout Inventory – General Survey” (MBI-GS; Schaufeli et al., 1996; German translation: Cillien et al., 2006) to assess emotional exhaustion. The subscale consists of five items which reflect the stress dimension of burnout and assess feelings of being emotionally overextended and exhausted by one’s work. For example, one item was: “I feel used up at the end of the work day.” All items are scored on a on a 6-point frequency rating scale ranging from 1 (“never”) to 6 (“very often”). Internal consistency was excellent (Cronbach’s α = 0.90).

#### Procedure

Initially, we devised the questionnaire together with several other researchers as part of a larger research project. Therefore, it comprised several items which were not relevant to this particular study.

We measured our dependent variable emotional exhaustion in a separate questionnaire (t2-questionnaire) that participants received nine to 10 weeks after filling in the first questionnaire measuring all other variables (t1-questionnaire).

Since we were a large group of researchers who all approached companies, organizations, and clubs alike, we allowed for several weeks during which the questionnaires could be answered. The first period, therefore, consisted of 9 weeks for the t1-questionnaire and the second one of 5 weeks for the t2-questionnaire. Depending on when we received the replies from the respective companies, we allowed for a break of nine to 10 weeks in between. We decided to use this relatively short time lag to counteract the frequent use of very long time lags that is typical for industrial and organizational psychology although it may identify cross-lagged effects only by chance (Dormann and Griffin, 2015).

## Results

To conduct the analyses, we used the programs R (R Development Core Team, 2017) and MPlus (Muthén and Muthén, 2019). Table 1 displays the means, standard deviations, and correlations of all variables on the individual level and Table 2 displays this information on the group level.

**TABLE 1:** Means, Standard Deviations, and Zero-Order Correlations of Variables (participant-level).

	***M***	***SD***	**1**	**2**	**3**
1. Perceived subgroups	4.07	1.38			
2. Team identification	5.24	1.19	−0.43***		
3. Subgroup identification	3.42	1.65	0.49***	−0.44***	
4. Emotional exhaustion	2.64	0.97	–0.04	−0.21*	0.06

This table only comprises participant-level variables. Because faultline strength is measured on the team level, we did not include it in this table. *p < 0.05, ***p < 0.001.

**TABLE 2:** Means, Standard Deviations, and Zero-Order Correlations of Variables (group-level).

	***M***	***SD***	**1**	**2**	**3**	**4**
1. Perceived subgroups	4.07	1.38				
2. Team identification	5.24	1.19	−0.37**			
3. Subgroup identification	3.42	1.65	0.52***	−0.28^†^		
4. Emotional exhaustion	2.64	0.97	0.00	–0.02	–0.09	
5. Faultline strength	0.41	0.21	0.06	0.30*	0.09	0.23

^†^*p* < 0.10, *p < 0.05, **p < 0.01, ***p < 0.001.

First, we analyzed the intraclass correlations (ICCs) of team identification and emotional exhaustion to examine how much the predicted variables depend on the team membership based on the complete dataset. ICC(1) reflects the amount of total variance of a measure that is explained by group membership, whereas ICC(2) indicates the homogeneity of the measure within the groups. For team identification, we found ICC(1) = 0.19 and ICC(2) = 0.35 and for emotional exhaustion, ICC(1) = 0.06 and ICC(2) = 0.15. Because of the hierarchical structure of our sample (i.e., participants nested within teams) and the amount of variance explained by group membership, we decided to conduct the analyses with a multilevel approach.

Subsequently, following the procedure outlined by Bliese (2016), we identified the random effect structure of the models using emotional exhaustion as dependent variable. Using perceived subgroups as focal level-1 predictor, we tested whether a multilevel model with a random intercept and slope fitted the data better than a model with just a random intercept. Because this was not the case, χ(2) = 2.37, *p* = 0.31, we conducted all multilevel analyses with random-intercept models with fixed slopes.

### Team Identification as a Mediator

Due to the hierarchical structure of our data and the nature of our hypotheses, we used a multilevel path model (that is, multilevel structural equation models with manifest variables only^3^) to test our hypotheses. The hierarchical structure of the sample and thus the multilevel approach analysis enabled us to separate the variance within a team from the variance between teams. Specifically, we constructed a multivelel path model on the basis of Hypotheses 1a and 1b. It contained emotional exhaustion – both on the within-team- and the between-team level as dependent variable –, and team identification with its within-team and between-team variance components as mediator. We used perceived subgroups with its within-team and between-team variance components as predictor on the individual and on the team level, and objective team-level faultline strength as a predictor on the team level. Team-level objective faultline strength and team-level perceived subgroups were set to correlate freely. On the team level, we included team size and numbers of subgroups as control variables. On the individual level, we controlled for age, educational background, and gender. The results of all models held when running the models without the control variables.

**FIGURE 2:**
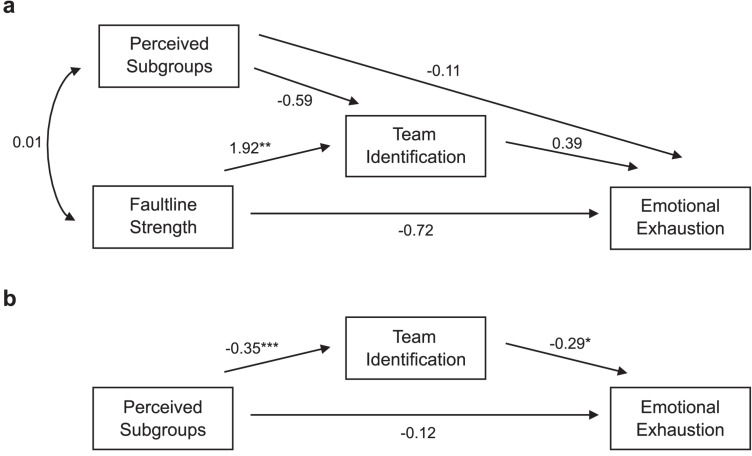
**(a)** Between and **(b)** within effects of the tested multilevel path model for Hypotheses 1a and 1b. Note that for the sake of clarity, the control variables are omitted from this diagram although they were included in the analysis. *p < 0.05, **p < 0.01, ***p < 0.001.

We fitted the model with the MPlus software (Version 8.4; Muthén and Muthén, 2019) with the analysis type TWOLEVEL and the maximum likelihood estimator. We specified two indirect effects corresponding to the two mediations proposed in Hypotheses 1a and 1b. The final model with unstandardized path coefficients is given in Figure 2.

Overall, on the basis of the criteria by Schermelleh-Engel et al. (2003), the model fitted to the data well, χ^2^(7) = 8.39, *p* = 0.30, RMSEA = 0.044, CFI = 0.960, TLI = 0.853, SRMR (within) = 0.021, SRMR (between) = 0.077. Hypothesis 1a stated that objective faultline strength is associated with emotional exhaustion via team identification. Contrary to the hypothesis, there was a positive main effect of objective team-level faultline strength on team identification, *b* = 1.92, SE = 0.62, *p* = 0.002, and no significant effect of the between-team mean levels of team identification on Emotional Exhaustion, *b* = 0.39, SE = 0.92, *p* = 0.669. Hypothesis 1a was therefore rejected by the data.

Next, we turn to perceived subgroups as a predictor. Of note, team-level objective faultline strength and team-level perceived subgroups were unrelated, *b* = 0.01, SE = 0.03, *p* = 0.711. Hypothesis 1b stated that team identification mediates the effect of perceived subgroups on emotional exhaustion. Because perceived subgroups is an individual level variable, we investigated the relationship on the individual level. As predicted, the level of perceived subgroups affected emotional exhaustion via team identification: Stronger perceived subgroups lead to weaker team identification, *b* = −0.35, SE = 0.09, *p* < 0.001, which, in turn, lead to stronger emotional exhaustion, *b* = −0.29, SE = 0.12, *p* = 0.014. The indirect effect also reached significance, *b* = 0.10, SE = 0.05, *p* = 0.038. Hypothesis 1b was thus supported by the data.

### Subgroup Identification as a Moderator

**FIGURE 3:**
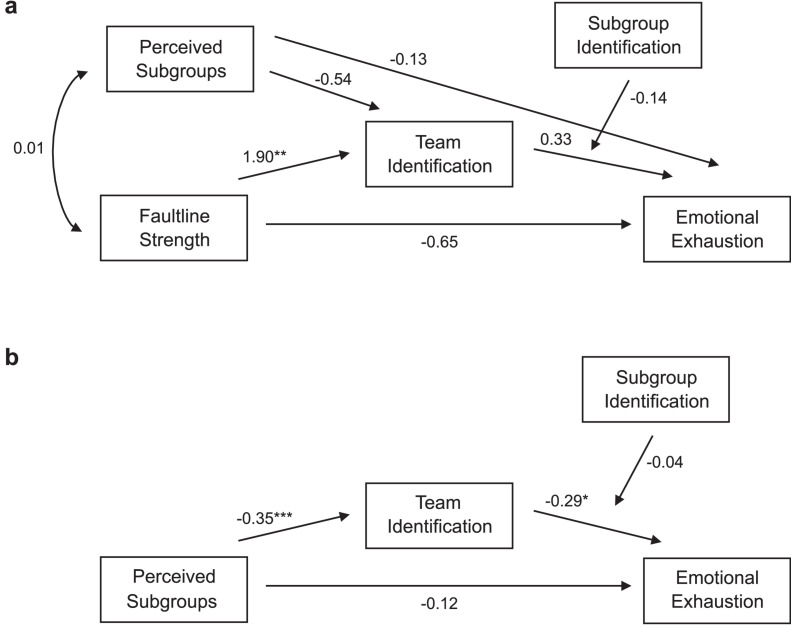
**(a)** Between and **(b)** within effects of the tested multilevel path model for Hypothesis 2. Note that for the sake of clarity, the control variables are not presented in this diagram although they were included in the analysis. *p < 0.05, **p < 0.01, ***p < 0.001.

Next, we analyzed the influence of subgroup identification in the effect of team identification on emotional exhaustion. In particular, Hypothesis 2 stated that subgroup identification moderates the effect of team identification on emotional exhaustion in a way that higher subgroup identification weakens the relationship between team identification and emotional exhaustion. To test it, we added an according interaction effect to the previous model as displayed in Figure 3. Based on the criteria by Schermelleh-Engel et al. (2003), this model did not fit to the data well, χ^2^(19) = 53.240, *p* < 0.001, RMSEA = 0.132, CFI = 0.483, TLI = −0.278, SRMR (within) = 0.092, SRMR (between) = 0.143. Contrary to our expectations, the effect of team identification on emotional exhaustion was not affected by participants’ identification with the subgroup. The moderating effect of subgroup identification on the effect of team identification on emotional exhaustion was not significant, neither on the team level of the model, *b* = −0.14, *SE* = 1.13, *p* = 0.905, nor on the within level, *b* = −0.04, *SE* = 0.05, *p* = 0.405. Therefore, Hypothesis 2 was not supported.

## Discussion

This study aimed to investigate the effect of subgroup structures in work teams and their perception on emotional exhaustion as a dimension of burnout. Based on self-categorization theory and social identity theory, we proposed that there is a negative relation between faultlines, hypothetical dividing lines between subgroups, and team identification which, in turn, is positively related to emotional exhaustion. Further, we hypothesized analogously that the effect of the subjective perception of subgroups on emotional exhaustion is mediated by team identification. Finally, we proposed that the identification with the respective subgroup can also have positive effects on emotional exhaustion and hypothesized that subgroup identification moderates the effect of team identification on emotional exhaustion. In particular, we argued that higher subgroup identification leads to a weaker relationship of team identification and emotional exhaustion. To test our hypotheses, we conducted a two-wave survey with teams from diverse backgrounds, measuring all predicting variables at t1 and emotional exhaustion as outcome measure at t2.

The results from a multilevel modeling analysis only partially confirmed our hypotheses. Contrary to our expectations, there was no indirect association of faultline strength and emotional exhaustion. However, in line with Hypothesis 1b, there was a significant indirect relation of perceived subgroups and emotional exhaustion which was mediated by team identification. Lastly, we did not find that subgroup identification moderated the association of team identification and emotional exhaustion.

### The Effect of Faultlines

We assumed that the stronger the faultline, the lesser do team members identify with their team and the more they report emotional exhaustion. However, we found that the stronger the faultline, the more do team members identify with their team. This is a remarkable finding because faultlines typically lead to negative outcomes (Thatcher and Patel, 2012). Positive outcomes of faultlines are scarce and usually limited to very specific conditions (e.g., Bezrukova et al., 2010). Many of the studies that found positive effects investigated informational faultlines – faultlines that emerge from differences in task-relevant attributes like educational background, tenure, and functional background (Cooper et al., 2014). Typically, they argue that informational faultlines may be beneficial – or at least not harmful – because differences in informational attributes lead to greater overall cognitive resources of the team, and thus, may improve performance (Bezrukova et al., 2009). For example, strong informational faultlines lead to better firm performance under low environmental dynamism, high complexity, and high munificence (Cooper et al., 2014). However, in our study we neither investigated purely informational faultlines nor did we consider any specific conditions as moderators. Educational background is the only one of the three attributes we used to calculate faultline strength that is task-relevant/informational. Thus, previous insights on informational faultlines seem insufficient to explain why strong faultlines were not detrimental but rather beneficial for team identification in our study. Also, our finding is in conflict with previous studies which found no correlation of faultlines and team identification (Bezrukova et al., 2009; Jehn and Bezrukova, 2010).

Interestingly, there is one study with a positive outcome of strong faultlines that uses almost the same mix of social category characteristics and informational characteristics we used for faultline calculation and even considered an outcome deeply connected to emotional exhaustion: Strong faultlines based on education, tenure, age, and gender weaken the positive effect of perceived interpersonal injustice on psychological distress (Bezrukova et al., 2010). In contrast to our study, Bezrukova et al. (2010) incorporated faultlines as a moderator into their model. While they measured psychological distress in terms of anxiety and depression, we focused on the strongly work-related emotional exhaustion and investigated team identification as a mediator. Despite those conceptual differences, both studies provide evidence that potential positive outcomes of strong faultlines are not limited to differences in informational characteristics leading to greater overall cognitive resources and subsequent performance gains. Strong faultlines may also be beneficial for individual-level subjective perceptions and feelings – as we showed for team identification.

Although we found clear evidence for those beneficial effects and, thus, further support for the theoretical conceptualization of faultlines as “healthy divides” (Bezrukova et al., 2010), it remains unclear why and how faultlines exerted positive effects in our case. Possibly, being part of a clearly distinguishable subgroup is enjoyable for team members. Stronger faultlines could lead to increased social support and other positive group processes which, in turn, affect psychological health positively. But while Bezrukova et al. (2010) identified subgroup cooperation to be the source of those positive effects, we failed to show the importance of subgroup identification in our study. The positive impact on team identification in our study clearly pertains to the whole team, not the subgroups. In that sense, the results we obtained remain somewhat surprising and highly specific. More research with different combinations of attributes is needed clarify the relationship of faultlines and team identification, as well as subgroup identification and cooperation.

### Fautlines and Perceived Subgroups

Another objective of this study was to investigate whether objective dormant faultlines or rather the active subjective perception of faultlines affect team identification. Therefore, we included perceived subgroups besides faultline strength as a predictor in our models. A conspicuous result is the low correlation of our objective and subjective faultline measures. On the one hand, this finding could be attributed to the selected variables for the faultline algorithm or our non-validated measure for perceived subgroups. Possibly, other attributes such as personality or skills were more relevant for the formation and perception of subgroups than the objective attributes that we used to measure faultlines in our study. Deeper level social category characteristics as well as other informational characteristics such as team tenure might have been more salient to team members. Thus, the faultline we measured with the algorithm does not necessarily reflect the faultline that was salient to team members. On the other hand, the current finding is in accord with previous studies which found no direct relationships between objective and subjective faultline measures (Homan et al., 2010; Jehn and Bezrukova, 2010; but see Zanutto et al., 2011). Previous research suggests that there are moderators of the relation of objective and subjective faultlines such as diversity beliefs or group entitlement configuration (Homan et al., 2010; Jehn and Bezrukova, 2010). For example, one study found a significant relationship only in teams with negative diversity beliefs (Homan et al., 2010). Unfortunately, we did not measure moderators of the relation of faultline strength and perceived subgroups. This suggests that the correlation of objective and subjective faultline measure could have been significant for some teams with certain characteristics but that we were not able to test this. Therefore, future studies should include moderators to investigate the relation of objective and subjective subgroup formation.

### The Effect of Perceived Subgroups

When including perceived subgroups as a predictor in our path model, the hypothesis that perceived subgroups affects emotional exhaustion via team identification was supported. This finding is line with self-categorization theory (Turner et al., 1987) which emphasizes the subjective perception of social categories rather than the objective composition of team members. According to self-categorization theory and social identity theory (Tajfel and Turner, 1986; Turner et al., 1987), a split of the work team into several subgroups should decrease identification with the whole team due to intergroup bias. Although our findings support self-categorization theory and social identity theory, previous findings did not necessarily find a significant relation of perceived faultlines and team identification (Jehn and Bezrukova, 2010). However, that study was conducted with undergraduate students in a lab setting, which differs clearly from our approach. Further research should investigate the processes and moderators of the effect of perceived subgroups on team identification. On the other hand, the negative effect of team identification on emotional exhaustion is in line with previous empirical results (Onyett et al., 1997; Haslam et al., 2005, 2009; Cicero et al., 2007; Jimmieson et al., 2010). Based on prior research, we argue that this relationship may emerge due to more cooperation and social support given and received from team members which identify more strongly with their whole team which in turn leads to lower levels of emotional exhaustion (Levine et al., 2002, 2005; Haslam et al., 2005; Lin et al., 2017). Nevertheless, our study is the first one to test the effect of the perception of subgroups on emotional exhaustion via team identification in one model.

While the indirect effect of perceived subgroups through team identification was significant, there was no significant direct effect of perceived subgroups on emotional exhaustion in the presence or absence of the mediator. Whereas the prominent framework on mediation by Baron and Kenny (1986) requires a significant direct path for a mediation, more recently, other authors have argued that an indirect effect does not necessarily require a significant relationship of predictor and outcome variable, especially for more temporally distal mediations as in our two-wave study (Shrout and Bolger, 2002; Cerin and MacKinnon, 2009; Hayes, 2009). Two possible reasons could explain this pattern. First, especially in distal relationships of outcome and predictor the power to find a mediation is greater than to detect a direct effect. Possibly, we did not have sufficient power to detect the direct effect. Second, it is possible that multiple mediators exist which operate in opposing directions and cancel each other out (Hayes, 2009; Pitts et al., 2017). Therefore, there might be another mediator which we did not investigate but interferes with team identification as mediator. Either way, more research is needed to clarify the direct relationship of perceived subgroups and emotional exhaustion.

By including measures of objective faultlines as well as subjective perception of subgroups, we were able to compare the effects of objective faultlines and the perception of subgroups on team identification and emotional exhaustion. While we did not find support for an effect of faultline strength on emotional exhaustion through team identification in the proposed direction, we found the hypothesized significant indirect effect of perceived subgroups. Our results emphasize the crucial role of subjective perceptions of group constellations for team processes. This is in line with not only self-categorization theory but also with previous findings which compared the effects of objective and subjective faultlines as well as diversity measures. In these studies, the perception of faultlines and diversity had a greater impact on team identification and other outcomes than the objective group composition (van Dick et al., 2008; Jehn and Bezrukova, 2010; Hentschel et al., 2013).

We proposed that faultlines and perceived subgroups are demands within the job demand-resources model of burnout (Demerouti et al., 2001). So far, to the best of our knowledge research has not considered subgroup structure as a job demand which could increase the risk of burnout. Our study does not support that faultlines *per se* pose a demand – on the contrary, our results rather point toward faultlines being a resource. If subgroup splits are subjectively perceived, however, they can foster emotional exhaustion. Accordingly, perception of subgroups can be considered as job demand within the job demand-resources model. In sum, it is more important for psychological health how people perceive their group environment than how the team is actually composed.

### Subgroup Identification

To the best of our knowledge, we were the first to differentiate team identification and subgroup identification as two related but distinct constructs in our study. We observed a negative correlation that should have been higher if both, team identification and subgroup identification, were the opposing ends of one continuum^4^. Therefore, our results oppose studies on diversity and team identification which thought of team identification as a one-dimensional construct that includes high subgroup identification as the lower end of a team identification scale (e.g., Hentschel et al., 2013). Instead, our observations are in line with cross-categorization theory (Brewer, 2000; Crisp and Hewstone, 2000). “Subgroup and team memberships can be viewed as a special sort of crossed categorization […]” (Bezrukova et al., 2009, p. 40): While it is possible that one identifies with a subgroup only and at the expense of identification on the team level, it is also possible that one strongly identifies with a subgroup but also identifies with the team. The latter is more likely, if there are cross-cutting categories enabling team members to see others as in-group and out-group members simultaneously (Hewstone et al., 2002).

However, we could not find support for the hypothesized attenuating effect of subgroup identification. Consequently, team identification seems to be the more powerful construct and subgroup identification seems not to have additional value to explain emotional exhaustion. Although subgroup members might benefit from their belongingness to a subgroup, these benefits might be offset by detrimental processes between subgroups (Hogg and Terry, 2000; Carton and Cummings, 2012).

Our rationale for hypothesizing that subgroup identification could be beneficial was based on the assumption that subgroup identification would result in similar positive outcomes as team identification. Based on the established relationships of team identification with team cooperation (Lin et al., 2017) and social support (Haslam et al., 2005), we assumed that subgroup identification is a prerequisite for cooperation and social support among subgroup members. It might be that we could not find the moderating effect of subgroup identification because high subgroup identification did not translate into strong cooperation and social support within the subgroups. Several factors that we did not consider in our study might have contributed to missing supportive behavior in subgroups. The theory of subgroups (Carton and Cummings, 2012) differentiates between three types of subgroups: identity-based subgroups, resource-based subgroups and knowledge-based subgroups. While we focused on differences in aspects of identity between subgroups, it is possible that these identity-based subgroups concomitantly differed in resources and knowledge. If subgroup existence is not or not exclusively based on social identity but also reflects differences in access to resources and knowledge, these differences may impair the ability to offer support within a subgroup. In this case, subgroup identification would have less power to enfold positive effects.

### Limitations

Our study goes beyond most of previous research in diversity and faultline research, using a two-wave design with two times of measurement. Furthermore, this study uses a sample including work teams of different companies in different domains as well as teams in voluntary work and clubs. Thus, the findings of our study are representative for a variety of teams. However, there are some limitations regarding the sample and the measurements. First of all, while the heterogeneity of the sample in terms of representativeness benefits the generalizability of the conclusions, at the same time, it also poses a downside. A heterogeneous sample makes it harder to find an effect due to more confounding variables. Therefore, potentially we could have been unable to find small effects. However, this also suggests that the effects that we found are generalizable to other samples. Second, due to the drop-out from t1 to t2, the measurement of emotional exhaustion relies on a relatively small sample, which reduces the interpretability of the results. Third, our sample also comprised relatively small teams (e.g., teams of three people). For those, the measurement of the formation and perception of subgroups could have been difficult because such groups would necessarily have to split up into subgroups comprising a single person. If the faultline algorithm computes a subgroup comprising a single person, measuring subgroup identification for this person is problematic and of limited use.

Furthermore, the measurement of the constructs is subject to discussion. One explanation for failing to find a moderating effect of subgroup identification could be the specially developed scale with weak psychometric properties (i.e., Cronbach’s α). In addition, despite evidence for the quality of the faultline algorithm and its predictive power (Meyer and Glenz, 2013), one could argue that in general an algorithm is not able to measure a team structure and is thus not appropriate to measure objective faultlines. Further, we based the faultlines on the demographic variables gender, age, and educational background which is in line with previous research (Thatcher and Patel, 2012). However, one may criticize that demographic variables are not suitable to describe faultlines within in a team and that other, perhaps psychological, variables are more meaningful for the formation of subgroups. Also, our measure of subgroup perception did not assess which variables the perception of subgroups is based on. Therefore, it is not clear whether the subgroup perception scale measured the same subgroups the algorithm identified or if the objective and subjective subgroup measures referred to differing subgroups.

### Implications

Despite those limitations, our study led to some interesting results with implications for further research and practical work. Although we failed to demonstrate an attenuating effect of subgroup identification, we still believe subgroup identification is a promising construct for further investigation. The correlation between subgroup identification and team identification in our sample supports our assumption that there is no one-dimensional construct of team identification with subgroup identification being one end of the continuum. If it was one-dimensional, we should have observed a higher negative correlation between team identification and subgroup identification. Thus, we rather believe that both, team identification and subgroup identification, are two distinct, though correlated variables. However, we need to find further support for our assumption. For example, further research could seek to identify situations in which both, team identification and subgroup identification, are high at the same time.

Although there has been promising research which found positive effects of subgroup processes (Bezrukova et al., 2010), we did not find that subgroup identification enfolded positive effects on emotional exhaustion. As discussed before, an uneven distribution of resources among subgroups may interfere with supportive behavior in subgroups. Thus, it would be fruitful to investigate subgroup identification and social support within subgroups while controlling for the distribution of resources. Additionally, a replication of our study with larger teams would be helpful to assess the true importance of subgroup identification. In doing so, it becomes much less likely that the faultline algorithm produces subgroups with only one person. Beyond that, we suggest to create a version of the faultline algorithm that generally avoids one-person-subgroups. Supplementing the existing algorithms in this way would be particularly helpful for studies which investigate variables that pertain to subgroups, like ours. The additional algorithm would help to separate research on subgroups from research on mavericks.

Along with this multi-faceted call for future research, we also derive some practical implications from our study. We could not find a relationship between faultline strength and emotional exhaustion. This indicates that there may be no need for team leaders, managers, recruiters, and others to be concerned that a team composition that hypothetically favors splits into subgroups based on demographics may impair psychological well-being. Our results support the idea that the alignment of differences in age, gender, and educational background alone does not foster emotional exhaustion. Instead, our results indicate that the prevention of subgroup perception and the promotion of team identification help to minimize the risk of burnout.

In sum, we conclude that obvious objective differences might be given too much attention when we talk about increased diversity. It is not these differences themselves but the subgroup perception that poses a demand on team members and brings along negative effects for mental health. Thus, it seems most promising to direct new burnout prevention programs toward avoiding subgroup perceptions, for example through regular team building and team development measures. In contrast, simply limiting the diversity employees encounter among their work team members by striving after homogenous teams is no guarantee for avoiding subgroup perceptions. Thus, avoiding diverse teams is not a helpful strategy to minimize the risk of burnout.

## Data Availability Statement

The raw data supporting the conclusions of this article will be made available by the authors, without undue reservation.

## Ethics Statement

Ethical review and approval was not required for the study on human participants in accordance with the local legislation and institutional requirements. The patients/participants provided their written informed consent to participate in this study.

## Author Contributions

KET and SKS contributed equally to this work and thus share the first authorship. All authors contributed to the design, data collection, data analysis, and manuscript preparation.

## Conflict of Interest

The authors declare that the research was conducted in the absence of any commercial or financial relationships that could be construed as a potential conflict of interest.

## Footnotes

1 The latter two are sometimes called depersonalization and personal accomplishment (as a reversed scale; Maslach and Jackson, 1981).

2 For the analysis of hypothesis H2, one additional participant was excluded because of missing data, resulting in a final sample of *N* = 104.

3 The usable sample size of 105 participants prevented us from using latent variables in the analysis.

4 Note that when we included all participants who took part at t1 in our correlational analysis (*N* = 536), the correlation was considerably smaller (*r* = −0.32, *p* < 0.001).

## Appendix

The data reported in this manuscript was collected as part of a larger data collection. Findings from the data collection have been reported in separate manuscripts. The table below displays where each variable appears in each study.

**TABLE A1:** Variables assessed in the joint data collection and used in this and other publications.

**Variables in the dataset**	**Schulte et al. (2020)**	**Current article (Tiede et al., 2021)**
Faultline strength		X
Perceived subgroups	X	X
Team identification		X
Subgroup identification		X
Emotional exhaustion	X	X
Stress	X	
Intragroup conflict	X	
Psychological empowerment	X	
Task interdependence	X	
Age and gender	X	X
Educational background		X
Nationality		
Migration background		
Religion		
Care responsibilities		
Team information (Team size, team tenure, leadership function)		
Team satisfaction		
Psychological safety		
Inclusive leadership		
Social support		
Perceived diversity		
Willingness to work with asylum seekers		
Contact with asylum seekers		
Personality (Big 5)		

*An X indicates that the variable has been used in the respective publication.*

**TABLE A2:** Sample description and comparisons of (a) all participants who completed only the first questionnaire (*N* = 358) and (b) participants who completed both the first and the second questionnaire (*N* = 112).

	**First wave only: *M* (SD) or%**	**Both waves: *M* (SD) or%**	**First wave only vs. both waves**
Age (years)	33.4 (11.9)	37.3 (12.0)	*t*(172.45) = 2.93, *p* = 0.004
Gender (% female)	55.1	65.4	χ^2^ = 2.69, *p* = 0.101
Education (% university degree or more)	43.3	56.8	χ^2^ = 5.58, *p* = 0.018
Team identification	4.9 (1.4)	5.2 (1.3)	*t*(202.27) = 2.11, *p* = 0.036
Subgroup identification	3.5 (1.5)	3.5 (1.7)	*t*(173.60) = 0.23, *p* = 0.819
Perceived subgroups	4.3 (1.4)	4.1 (1.4)	*t*(183.30) = 0.89, *p* = 0.375
Emotional Exhaustion	2.7 (1.1)	2.7 (1.0)	*t*(199.81) = 0.42, *p* = 0.678
